# In Memoriam: The Enduring Influence of Nancy J. Cox (1948–2026)

**DOI:** 10.3201/eid3210.260830

**Published:** 2026-10

**Authors:** Daniel B. Jernigan, Jacueline M. Katz, Keiji Fukuda

**Affiliations:** Johns Hopkins Bloomberg School of Public Health (D. Jernigan); retired (J. Katz, K. Fukuda)

**Keywords:** Nancy J. Cox, influenza, CDC, Global Influenza Surveillance and Response System

In Italian usage, *influenza* refers broadly to influence, which Treccani defines as the “influence or action exerted by the stars on human character and destinies” (*1*). Nancy J. Cox, PhD (Figure), has had an enduring and tremendous influence on the global public health and influenza scientific communities. Over a distinguished career spanning nearly 4 decades, she advanced ideas, systems, and people that shaped contemporary influenza science and practice. Through her work in epidemiology, virology, outbreak response, prevention, vaccinology, surveillance, and One Health, her leadership and diplomacy influenced institutions worldwide. She was recognized with numerous awards and leaves a legacy that will continue to shape future generations of public health scientists.

**Figure:**
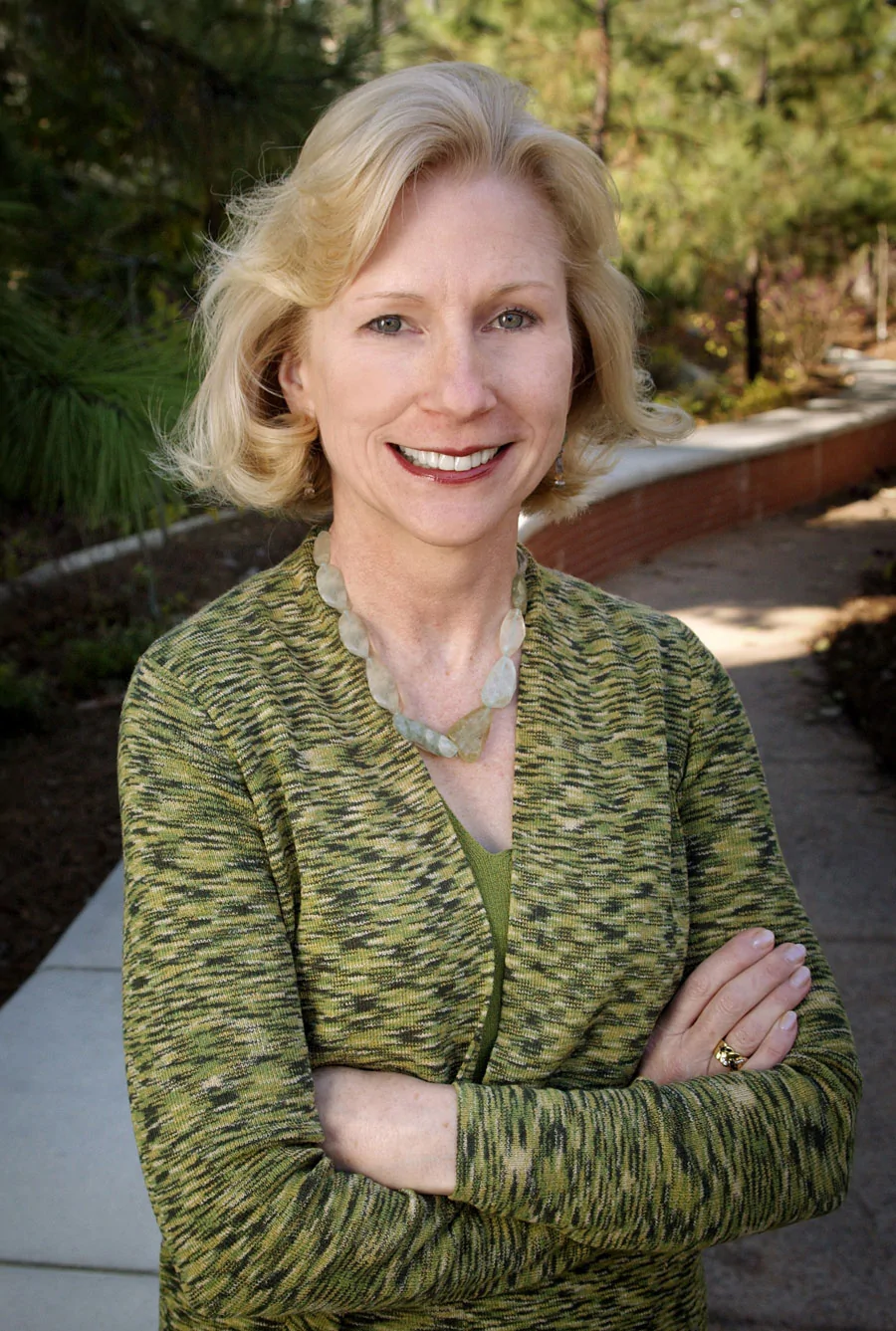
Nancy J. Cox (1948–2026) in June 2008. Photograph courtesy of the Centers for Disease Control and Prevention.

## Early Life and Scientific Foundations

Nancy was born in rural Iowa in 1948 and earned a National Science Foundation scholarship to Iowa State University, completing her degree with highest honors. She then attended Cambridge University as a Marshall Scholar and entered the influenza field in the Division of Virology, Department of Pathology at Cambridge. Her early work examined molecular aspects of influenza virus replication, a foundation that shaped the rest of her career (*2*).

Her focus on the fundamental biology of influenza viruses, especially how they evolved and interacted with the immune system, led her to understand how subtle genetic and antigenic differences affect epidemics. That insight contributed to one of her most enduring public health roles: guiding the selection of influenza viruses for use in the influenza vaccine. Nancy set the standards for how an influenza virus is characterized by using genomic and antigenic testing. As the head of a World Health Organization (WHO) Collaborating Center for Surveillance, Epidemiology, and Control of Influenza at the Centers for Disease Control and Prevention (CDC), she provided globally recognized leadership for the process used for preparing and selecting viruses for updating influenza vaccines.

Over 37 years at CDC, Nancy helped transform a small team of scientists into the preeminent global center for influenza surveillance, epidemiology, research, and preparedness. She authored more than 278 scientific publications and helped define 3 pillars of modern influenza work: quantifying disease burden, characterizing emerging viruses, and using global systems for surveillance and response.

There were 2 settings where Nancy was most comfortable in her professional life. The first was when she was diving deep into the testing of viruses. At weekly meetings, Nancy was always at the head of the table, often wearing a tailored suit jacket, and leading her staff as they reviewed detailed spreadsheets of virus test data. Such gatherings were an odd hybrid of cutting-edge biotechnology and priestly ritual, divining the future of each virus.

The other setting was at small international meetings, where Nancy would engage in deep conversations on virus protein structure with colleagues during the day and then stay up late with the same friends sharing stories and recollections. Nancy understood that the best scientific collaborations were built not only on shared questions and rigorous methods but also on trust, respect, generosity, and genuine care for the people doing the work.

## Translating Virology into Public Health Action

Nancy could see the connections between what a virus was doing in the laboratory and how those findings might affect global vaccine policy. She knew the science and she understood the politics. She could see how it all came together, from the genome in the laboratory to the geopolitical in Geneva. She integrated information across disciplines, spanning virus detection to disease prevention, and made it accessible and relevant for a comprehensive global influenza intelligence system.

Nancy loved having things well-categorized, appropriately named, and understandable. She led the development of the Influenza Risk Assessment Tool, a structured framework for integrating virologic, epidemiologic, and population-level data to compare the pandemic potential of novel influenza A viruses (*3*). That framework has guided decision-making for novel influenza A viruses and reflects one of her signature approaches: moving from “what is this virus?” to “what should the world do about it?”

## Architect of Global Influenza Surveillance and Vaccine Virus Selection

In 1992, Nancy became chief of CDC’s Influenza Branch and Director of the WHO Collaborating Center for Surveillance, Epidemiology, and Control of Influenza at CDC. In those roles, she strengthened WHO’s Global Influenza Surveillance and Response System (GISRS), a worldwide network of laboratories and public health institutions (*4*,*5*). Now comprising more than 160 National Influenza Centers in 136 countries and 7 Collaborating Centers, GISRS is the world’s main system for tracking influenza virus evolution in real time, selecting viruses for annual influenza vaccines and identifying viruses with pandemic potential (*6*).

Nancy foresaw the importance of China’s development of influenza monitoring capacities because of the role of China and other countries in the region in the emergence of new influenza virus variants. Collaborating with WHO, she helped expand GISRS and build surveillance and response capabilities in many countries; these systems later proved fundamental during the COVID-19 pandemic. Her efforts expanded the global footprint of the CDC Influenza Division’s technical and funding support to national laboratories all around the world. That investment grew substantially over time to fill a critical gap in global surveillance and influenza prevention programs, and created an enduring trust of global partners in CDC, something Nancy championed as a requirement for collaborative responses to influenza threats.

She also oversaw establishment of the Influenza Reagent Resource (later named the International Reagent Resource) to support manufacturing, stockpiling, and distribution of diagnostic reagents and test kits globally. This service fundamentally changed global surveillance by removing costs of core reagents and materials as a roadblock to robust surveillance systems.

Nancy advanced modernization of influenza virus characterization methods. She promoted a “Seq-First” approach, in which genomic sequencing precedes traditional antigenic and serologic testing, enabling laboratories to expand evaluation of new variants for possible incorporation into seasonal influenza vaccines. Her focus on annual global and national vaccine strain selection, in partnership with WHO and the US Food and Drug Administration, was a key contribution to public health.

Richard Hatchett, chief executive officer of the Coalition for Epidemic Preparedness Innovations, stated, “Nancy was a giant and a tremendous advocate for science, public health, and international partnership. She played a huge role in establishing the international surveillance networks that protect us all, and her dedication to her work and to public service was immense.”

## Responding to Emerging Threats

Nancy’s career spanned the emergence of highly pathogenic avian influenza A(H5N1) in 1997 (*7*), the 2009 influenza A(H1N1) pandemic (*8*), and influenza A(H7N9) in China in 2013 (*9*). In those events, she helped define how animal influenza viruses can cross species barriers to humans, leading her to emphasize integration of animal and human surveillance, an approach central to contemporary One Health strategies.

During the 2009 H1N1 pandemic, she guided US response policies, providing steadiness, clarity, and confidence, and serving as a trusted voice for public and private stakeholders. Her dedication and persistence shone clearly when, early in the pandemic response, her home was struck by lightning and burned down. When most people would have been near a breaking point, Nancy was still driving the response forward, focused on the outcome rather than the obstacle.

## Building Bridges through Influenza Diplomacy

Nancy believed in, and exemplified, CDC’s longstanding role of assisting countries around the world. She also believed that the United States and the international community were best served when scientific and public health knowledge, experience, and support were shared. 

She was recognized internationally as a uniquely authoritative and trusted influenza expert. WHO frequently sought her wisdom and assistance for critical activities, including development and evaluation of new Collaborating Centers, Pandemic Influenza Preparedness Framework negotiations, and international missions to assess complex situations such as influenza A(H7N9) in China and influenza A(H5N1) in Egypt.

Dr. Margaret Chan, former director-general of WHO, wrote: “Your commitment, dedication, scientific attitude and quality of work has inspired everyone in the influenza community and has set an excellent example for the public health. We will miss your brilliant insights, unmatchable expertise, strong leadership and diplomatic skills, as well as your energy, dynamism and grace in our continuous defense against influenza threat.”

## Mentor and Advocate

Nancy was a mentor and inspiration to scientists within her own team and around the world. Her scientific excellence, tireless work ethic, and collegial attitude inspired respect and admiration and provided a role model for young and senior scientists alike.

Dr. Rebecca J. Garten Kondor, who has taken on the important role of guiding vaccine virus selection in CDC’s Influenza Division, recalled, “Early in my career I was fortunate to benefit from Nancy’s leadership and mentorship, where I learned the value of bringing multiple disciplines to the table and setting high standards of scientific rigor and integrity. Getting it right—and having confidence in our results—is essential because CDC science is widely respected, and sharing our analysis capabilities and know-how with global partners strengthens influenza surveillance and pandemic preparedness.”

Professor Yoshi Kawaoka stated, “So much of my career has been influenced by your work. You leave behind a wonderful footprint — you have touched the lives of and will be missed by many.” Professor Guan Yi, University of Hong Kong, China, called her “a scientist of extraordinary vision, a tireless diplomat of public health, and a deeply cherished friend and mentor to so many of us in the global influenza research community.”

Upon Nancy’s retirement in 2014, then-Senator Tom Harkin (D-Iowa) honored her by stating in the Congressional Record, “During her 37 years at CDC, Dr. Cox has served as mentor, educator, supervisor, and supporter to hundreds of individuals: undergraduates, medical and PhD students, postdoctoral fellows, laboratory and epidemiology staff members, journalists, and visiting researchers” (*10*).

## Recognition and Enduring Legacy

Nancy was referred to as a “walking encyclopedia of influenza,” and her knowledge of influenza was legendary. She fostered a culture of high quality, scientific excellence, mutual respect, and accountability, and led through example. One former colleague said, “She held our feet to scientific fire, only because she could walk across those coals.” Nancy spent her career raising the bar for scientific excellence, an approach needed now more than ever.

Nancy received numerous awards, including the Samuel J. Heyman Service to America Medal as Federal Employee of the Year in 2006 and recognition by Time magazine as one of the 100 most influential people of 2006. Dr. Anthony Fauci, former Director of the National Institute of Allergy and Infectious Diseases at the National Institutes of Health, considered her “one of the leading figures in the influenza research community,” whose work “benefitted millions of Americans and people around the world.”

Professionally, her legacy is reflected in the systems she helped build: global surveillance networks, vaccine selection processes, and preparedness frameworks that continue to protect populations worldwide. Personally, among those privileged to know and work with her, the deepest impression is her legacy of warmth, generosity, fairness, inclusiveness, and commitment to public health.

Dr. Nancy Cox died on April 23, 2026. She is survived by her husband, daughter, her granddaughter, stepson and wife, three step-grandchildren, and other family members.
